# Identification of a novel AMPK-PEA15 axis in the anoikis-resistant growth of mammary cells

**DOI:** 10.1186/s13058-014-0420-z

**Published:** 2014-08-06

**Authors:** Sravanth K Hindupur, Sai A Balaji, Meera Saxena, Shubham Pandey, Gopalkrishnashetty Sreenivasmurthy Sravan, Namrata Heda, M Vijaya Kumar, Geetashree Mukherjee, Devaveena Dey, Annapoorni Rangarajan

**Affiliations:** 1 Department of Molecular Reproduction, Development and Genetics, Indian Institute of Science, CV Raman Road, Bangalore, 560012 India; 2 Department of Pathology, Kidwai Memorial Institute of Oncology, Hosur Road, Bangalore, 560029 India

## Abstract

**Introduction:**

Matrix detachment triggers anoikis, a form of apoptosis, in most normal epithelial cells, while acquisition of anoikis resistance is a prime requisite for solid tumor growth. Of note, recent studies have revealed that a small population of normal human mammary epithelial cells (HMECs) survive in suspension and generate multicellular spheroids termed ‘mammospheres’. Therefore, understanding how normal HMECs overcome anoikis may provide insights into breast cancer initiation and progression.

**Methods:**

Primary breast tissue-derived normal HMECs were grown as adherent monolayers or mammospheres. The status of AMP-activated protein kinase (AMPK) and PEA15 signaling was investigated by immunoblotting. Pharmacological agents and an RNA interference (RNAi) approach were employed to gauge their roles in mammosphere formation. Immunoprecipitation and *in vitro* kinase assays were undertaken to evaluate interactions between AMPK and PEA15. *In vitro* sphere formation and tumor xenograft assays were performed to understand their roles in tumorigenicity.

**Results:**

In this study, we show that mammosphere formation by normal HMECs is accompanied with an increase in AMPK activity. Inhibition or knockdown of AMPK impaired mammosphere formation. Concomitant with AMPK activation, we detected increased Ser^116^ phosphorylation of PEA15, which promotes its anti-apoptotic functions. Inhibition or knockdown of AMPK impaired PEA15 Ser^116^ phosphorylation and increased apoptosis. Knockdown of PEA15, or overexpression of the nonphosphorylatable S116A mutant of PEA15, also abrogated mammosphere formation. We further demonstrate that AMPK directly interacts with and phosphorylates PEA15 at Ser^116^ residue, thus identifying PEA15 as a novel AMPK substrate. Together, these data revealed that AMPK activation facilitates mammosphere formation by inhibition of apoptosis, at least in part, through Ser^116^ phosphorylation of PEA15. Since anoikis resistance plays a critical role in solid tumor growth, we investigated the relevance of these findings in the context of breast cancer. Significantly, we show that the AMPK-PEA15 axis plays an important role in the anchorage-independent growth of breast cancer cells both *in vitro* and *in vivo*.

**Conclusions:**

Our study identifies a novel AMPK-PEA15 signaling axis in the anchorage-independent growth of both normal and cancerous mammary epithelial cells, suggesting that breast cancer cells may employ mechanisms of anoikis resistance already inherent within a subset of normal HMECs. Thus, targeting the AMPK-PEA15 axis might prevent breast cancer dissemination and metastasis.

**Electronic supplementary material:**

The online version of this article (doi:10.1186/s13058-014-0420-z) contains supplementary material, which is available to authorized users.

## Introduction

Epithelial cells grow attached to their extracellular matrix (ECM) composed of collagen, elastins, fibronectin, laminin, proteoglycans and glycosaminoglycans [1]. Signals emanating from cell-matrix interactions regulate gene expression, cell growth and survival [2]. Consequently, matrix withdrawal triggers a form of apoptosis, termed anoikis, in normal adherent (ADH) cells [3]. Anoikis plays an important role during development as well as in the maintenance of tissue homeostasis in adult life [4]. In contrast, acquisition of anoikis resistance is a hallmark of epithelial cancer progression [5].

Untransformed mammary epithelial cells undergo anoikis upon matrix detachment [6]. Intriguingly, recent studies have demonstrated that a small population of normal human mammary epithelial cells (HMECs) survive under anchorage-independent conditions to generate floating spheroids termed ‘mammospheres’ (MS) when cultured in ultra-low (UL) attachment plates under serum-free conditions [7],[8]. However, the mechanisms that enable this subset of normal HMECs to escape anoikis and survive in suspension remain unexplored. Acquisition of anoikis resistance is considered to be a prerequisite for solid tumor metastasis, enabling cancer cells to survive in the circulation [9]. Moreover, in the context of mammary gland, anoikis resistance is thought to be involved in luminal filling associated with early breast lesions such as ductal carcinoma *in situ*[10]. Therefore, understanding the molecular mechanisms that enable a subset of normal HMECs to withstand anoikis and generate MS is likely to provide important insights into normal mammary gland biology, as well as breast cancer progression.

The AMP-activated protein kinase (AMPK) is a cellular energy sensor that is activated under stresses leading to an increase in the AMP: *adenosine triphosphate* (ATP) ratio, such as, nutrient deprivation, hypoxia, oxidative stress and endoplasmic reticulum (ER) stress [11]. Mammalian AMPK is a heterotrimeric complex consisting of one catalytic subunit α (63 kDa) and two regulatory subunits β and γ (38 and 36 kDa, respectively); each of these subunits has multiple isoforms (α1 and α2, β1 and β2, γ1, γ2, and γ3). AMP binds to the γ subunit of AMPK and brings about its allosteric activation. Additionally, AMPK is phosphorylated at Thr^172^ within its α subunit by liver kinase B1 (LKB1), Ca^2+^/calmodulin-dependent protein kinase kinase (CaMKK) and transforming growth factor-β activated kinase (TAK), and this phosphorylation is essential for its functional activation [11]. Recent reports have highlighted the importance of AMPK signaling in the survival of both normal and cancer cells under metabolic stress conditions [12],[13]. We have demonstrated that AMPK activation can protect cancer cells from glucose deprivation-induced stress by inducing autophagy [14], an evolutionarily conserved cellular catabolic process. Recently, AMPK activation has also been implicated in anoikis resistance [15]-[17]. Also, increased levels of phosphorylated (p)AMPK was reported in MCF10A immortalized mammary epithelial cells subjected to matrix detachment [18], suggesting that AMPK signaling may be involved in the survival and growth of HMECs in suspension.

The phosphoprotein enriched in astrocytes 15 kDa/phosphoprotein enriched in diabetes (PEA15/PED) is a multifunctional protein highly expressed in astrocytes [19]. PEA15 plays a critical role in restricting extracellular signal-regulated kinase (ERK) in the cytoplasm [20], thus functioning as a tumor suppressor. However, recent studies have shown that phosphorylation leads to a change in its binding partners and cellular functions [21]. Phosphorylation at its Ser^116^ position has been shown to promote its anti-apoptotic functions by binding to Fas-associated death domain protein (FADD) and preventing the recruitment of initiator caspases [22]. More recently, PEA15 phosphorylated at Ser^116^ has been shown to protect glioma cells from glucose deprivation-induced apoptosis [23]. Thus, phosphorylated PEA15 can provide anti-apoptotic signals under stress conditions.

In this study, we explored the signaling mechanisms that enable the anoikis-resistant outgrowth of MS. We show that MS formation is associated with an increase in AMPK activity, and this activation is essential for sphere formation. We further show that AMPK facilitates MS formation by inhibiting apoptosis, at least in part, through phosphorylation of PEA15 at Ser^116^ residue. We additionally show that this AMPK-PEA15 signaling axis is also associated with the anchorage-independent growth of breast cancer cells *in vitro* and *in vivo*, suggesting that cancer cells may exploit properties inherent within a subset of normal cells for their survival during matrix-deprived conditions. Thus, our data provides support for the protumorigenic functions of AMPK, and uncovers a novel AMPK-PEA15 axis in the anoikis-resistant growth of mammary epithelial cells.

## Methods

### Primary HMEC culture and mammosphere formation

Primary breast tissues were procured from Kidwai Memorial Institute of Oncology (KMIO) Bangalore, India, as approved by the Medical Ethics Committee (the institutional review board (IRB) of KMIO) and in compliance with the ethical guidelines of the Indian Institute of Science (IISc). Patient consent was obtained in a written form prior to surgery, as per the protocol approved by the IRB of KMIO. Primary breast tissue was processed as described previously [8]. In brief, mechanical followed by enzymatic processing of normal breast tissue yielded organoids, which were trypsinized and filtered through a 70 μm cell strainer (BD Biosciences, San Jose, CA, USA) to obtain largely single cells. These freshly derived HMECs were cultured in serum-free media containing 10 ng/ml human epidermal growth factor (hEGF), 1 μg/ml hydrocortisone, 10 μg/ml insulin, 4 ng/ml heparin and B27 at a density of 2 × 10^5^ cells per 2 mL/well of 6-well UL attachment plates (Corning Inc., New York, NY, USA). Under these conditions primary MS were formed within a week.

**Figure 1:**
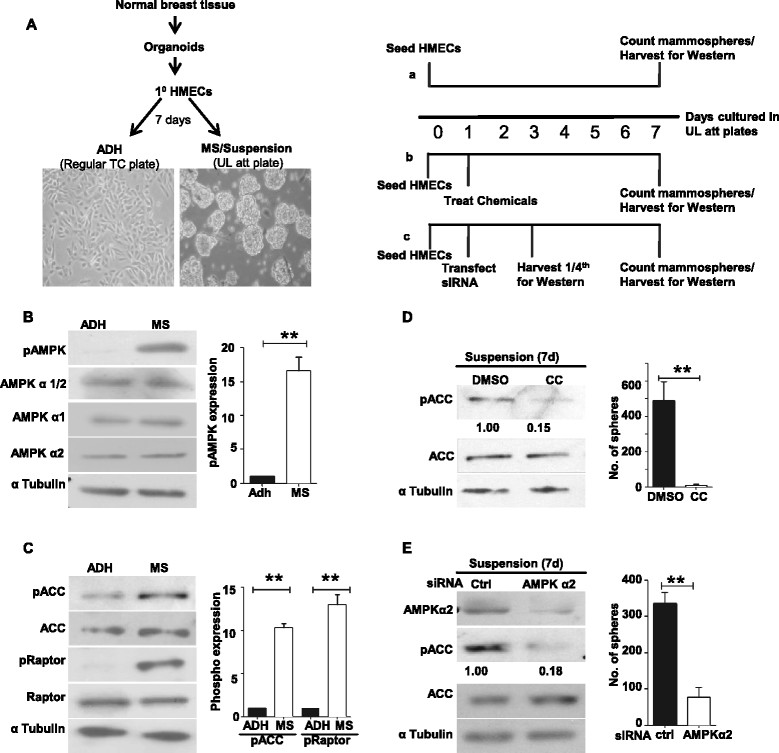
**Mammospheres show elevated AMP-activated protein kinase (AMPK) activity. (A)** Phase-contrast microscopic images (left) of freshly isolated primary human mammary epithelial cells (HMECs) cultured for a week as monolayers in adherent (ADH) condition in regular tissue culture (TC) dishes, or as mammospheres (MS) in suspension in ultra-low attachment plates (UL plates); magnification ×200. Experimental strategies (right) involving HMECs seeded in suspension (also see Methods). **(B** and **C)** Primary HMECs cultured in ADH condition or in suspension as MS were harvested at the end of a week and subjected to immunoblot analyses for the specified proteins. Graphs represent densitometry analyses of western blots to quantify phosphoprotein relative to total protein. Error bars represent standard error of the mean (SEM), (n = 6). The phosphorylated (p)AMPK antibody recognizes both AMPK α1 and α2 isoforms; antibodies against total AMPK recognize both α1/2 together, or only individual isoforms α1 or α2; α-tubulin served as loading control for all western blot experiments. **(D)** Primary HMECs were cultured in UL plates in the presence of 10 μM AMPK inhibitor, Compound C (CC); dimethyl sulfoxide (DMSO) served as vehicle control. Two days after treatment, a quarter of the cells were harvested and subjected to immunoblot analyses for specified proteins, while the rest were scored for total number of MS formed at the end of a week (graph). Error bar represents SEM, (n = 4). **(E)**. Primary HMECs seeded in UL plates were transfected with control small interfering RNA (siRNA) or siRNA targeting AMPK α2 (Dharmacon, Thermo Fisher Scientific, Waltham, MA, USA). Two days posttransfection, a quarter of the cells were harvested and subjected to immunoblot analyses for the specified proteins, while the rest were scored for total number of MS formed at the end of a week (graph). Error bars represent SEM; n = 4.

To compare signaling in adherent versus suspension conditions, freshly derived HMECs were typically seeded in parallel in conventional 9 cm tissue culture plates for adherent (ADH) culture or in 6-well UL attachment plates for MS formation (Figure 1). Cells were harvested at the end of seven days for further experimentation, or as indicated (Figure 1). Total numbers of spheres formed were counted at the end of seven days under a microscope as described previously [8].

### Western blotting and immunoprecipitation

Whole cell lysates for western blotting were prepared with lysis buffer containing 1% NP40 detergent, 0.5% sodium deoxycholate, 0.1% SDS, 50 mM sodium fluoride, 1 mM sodium orthovandate, 10 mM sodium pyrophosphate (Sigma-Aldrich, St Louis, MO, USA) and protease inhibitors (Roche, Basel, Switzerland). Protein concentration was estimated with Bradford reagent and equal amount of protein was resolved by SDS-PAGE using Bio-Rad apparatus, (Bio-Rad Laboratories, Hercules, CA, USA) transferred to a PVDF membrane (EMD Millipore, Merck, Darmstadt, Germany) and probed with appropriate antibodies. Horseradish peroxide (HRP)-coupled secondary antibodies were obtained from The Jackson Laboratory (Bar Harbor, ME, USA), and immunoblots were visualized using PICO reagent (Thermo Fisher Scientific, Waltham, MA, USA). Primary antibodies against pAMPK, total AMPK, phosphorylated acetyl-Co-A carboxylase (pACC), total ACC, pRaptor, total Raptor, cleaved poly(ADP-ribose) polymerase (PARP), caspase-3 and caspase-8 were purchased from Cell Signaling Technology (Danvers, MA, USA). The antibodies against total PEA15 and pPEA15 Ser^104^ were from Cell Signaling Technology, while pPEA15 Ser^116^ was from Thermo Fisher Scientific. The levels of respective total proteins was probed and observed to be the same for all western blots involving phosphoprotein analyses. Anti α-tubulin (Calbiochem, Merck, Darmstadt, Germany) served as the loading control in all western blots. Multi-panel blots in a given figure were assembled by reprobing the same blot. For densitometry analyses of western blots, band intensities acquired using Image-J were first normalized to tubulin expression, and the ratio of phosphoprotein relative to its total protein is represented graphically as fold change over control condition.

For immunoprecipitation (IP) assays, cells were lysed with co-IP lysis buffer (Pierce co-IP Kit 26149; Thermo Fisher Scientific). One milligram of total proteins was cleared with control agarose resin for 1 hr at 4°C. The cleared supernatant was transferred to spin columns containing agarose resin conjugated with 20 μg of specified antibodies. Incubation was done overnight at 4°C, with gentle rocking. For control co-IP, lysates were added to spin columns containing agarose resin conjugated to 20 μg immunoglobulin G (IgG) (Invitrogen, Carlsbad, CA, USA) and agarose resin alone. The antibody-antigen complexes were given six washes with lysis buffer and eluted by incubating the antigen-antibody complex with 50 μl low pH elution buffer provided in the kit. The eluates were resuspended in SDS sample buffer, boiled for 3 min and analyzed by SDS-PAGE and subsequent western blots.

### Pharmacological chemicals inhibitors/activators

Pharmacological chemicals used in this study include AMPK activator A769662 (100 μM [24], University of Dundee, UK), AMPK inhibitor Compound C (6-[4-(2-piperidin-1-ylethoxy-phenyl)]-3-pyridin-4-yl-pyrrazolo [1,5-a]-pyrimidine (10 μM [25], Calbiochem) and LY294002 (10 μM [26], Cell Signaling Technology). Absolute dimethyl sulfoxide (DMSO; Calbiochem) was used as solvent to make stock solutions, which were typically diluted 1:1000 to obtain working dilutions; identical concentration of DMSO was used as vehicle control. Pharmacological agents were typically replenished every three days.

### RNAi-mediated knockdown experiments

Primary HMECs were transfected with Accell small interfering RNA (siRNA) oligos (GE Healthcare Dharmacon, Little Chalfont, UK) targeting AMPK α2 and PEA15 as per the manufacturer’s protocol; nontargeting pool siRNA (GE Healthcare Dharmacon) was used as control siRNA. Breast cancer cell lines were transfected with specific siRNA oligos targeting AMPK α2 and PEA15 (Cell Signaling Technology); universal negative siRNA (Sigma-Aldrich) was used as control. Adherent Michigan Cancer Foundation 7 breast cancer cell line (MCF7) and breast cancer cell line derived from metastatic site (pleural effusion) (MDAMB231) were transfected with 20 nM siRNA using oligofectamine (Invitrogen). After 48 hrs of siRNA transfection, the cells were trypsinized and seeded in methylcellulose for further experimentation. AMPK α2 knockdown stable cells were generated by transfecting MDAMB231 and BT 474 cells with a pool of four short hairpin RNA (shRNA) constructs targeting AMPK α2 (pRFP-C-RS-PRKAA2) or scrambled (HuSH-29 shRNA vectors; (Origene Technologies, Rockville, MD, USA) using Lipofectamine-2000 (Invitrogen). Stable cells were generated using puromycin (0.5 μg/ml) selection followed by flow cytometer-based sorting (MoFlo; Beckman Coulter, Brea, CA, USA) for RFP expression (encoded by the vector) and were expanded and frozen for future use. Knockdown was confirmed by immunoblotting.

### Lentiviral infection of primary HMECs and growth in methylcellulose

Lentiviruses were generated as described previously [27]. In brief, HEK293T cells were transfected with pCSCG lentiviral vectors encoding for wild-type (WT) and S116A mutant of PEA15 along with packaging vectors: pCMV-VSVG and pHR∆8.2. HMECs seeded in 6-well UL attachment plates were spin-infected with viral supernatant in the presence of protamine sulphate (8 μg/ml). Two days following infection, a quarter of the cells were harvested for immunoblot analyses for the specified proteins and the remaining cells were counted and subjected to mammosphere formation in methylcellulose (as described for cancer spheres below). Mammospheres formed by GFP (encoded by the CSCG vector) positive cells were scored at the end of seven days.

#### In vitro kinase assay

One microgram of purified PEA15 (Abcam, Cambridge, UK) was incubated in the presence or absence of 1 μg of AMPK heterotrimer (Cell Signaling Technology) for 30 min at 30°C in AMPK kinase buffer (Cell Signaling Technology) supplemented with 50 μM ATP and 0.1 mM AMP (Sigma-Aldrich). Reactions were terminated by the addition of SDS sample buffer, resolved by SDS-PAGE and analyzed by western blotting using phosphorylated (p)PEA15 Ser^116^ and total PEA15 antibodies. For radioactive *in vitro* kinase assay, 1 μg of purified PEA15 was incubated in the presence or absence of 1 μg of AMPK heterotrimer along with (γ32P) ATP (0.3 μCi) for 30 min at 30°C in AMPK kinase buffer (Cell Signaling Technology) supplemented with 50 μM ATP and 0.1 mM AMP. Reactions were terminated by the addition of SDS sample buffer. The product of the kinase assay was resolved by SDS-PAGE, the gel was dried exposed to a phosphoimager screen (GE Healthcare) for 4 hrs and scanned using a Typhoon 9210 scanner (GE Healthcare).

### Cell culture, plasmids, transfections, and stable cell lines

Breast cancer cell lines MCF7, BT474 and MDAMB231, and HEK 293 T (from the American Type Culture Collection (ATCC)) were cultured in Dulbecco’s modified Eagle’s medium (DMEM) containing 10% fetal bovine serum (FBS) and maintained in standard 5% CO_2_ incubator at 37°C. Authenticity of the cell lines used was evaluated and verified by Bio-Synthesis, Inc. (Lewisville, TX, USA) employing genotyping of 15 short tandem repeat (STR) loci and the amelogenin gene (AMEL), and comparison with genotype information at the ATCC. The myc-tagged human AMPK α2 pCMV-Tag 3B used in immunoprecipation experiments was a kind gift from Ronald Evans. Flag-tagged WT and S116A mutant of PEA15 were a kind gift from Dr. W. Roth. These were subcloned into the EcoR1 site of the CSCG lentiviral vector, and protein expression confirmed by western blotting. MCF7 cells were transiently transfected with CSCG-PEA15 constructs using Lipofectamine-2000 and seeded in methylcellulose to assess for sphere formation. MDAMB231 and BT474 stable cell lines expressing CSCG-WT or S116A mutant PEA15 were made by two rounds of GFP (encoded by the CSCG vector) sorting in MoFlo (Beckman Coulter, Danvers, MA, USA) and these stable cells were used for *in vitro* sphere formation experiment and *in vivo* tumorigenicity experiments.

### Sphere formation in methylcellulose

Primary HMECs infected with CSCG-PEA15 constructs were trypsinized, counted and resuspended in a 1.5% slurry of methyl cellulose (in DME-F12 supplemented with growth factors) and plated on 0.6% noble agar-coated plates at a density of 1 × 10^5^ cells/35 mm dish and grown for seven days.

Adherent MCF7 and MDAMB231 cells were trypsinized, counted and resuspended in a 1.5% slurry of methyl cellulose (in DMEM supplemented with 20% FBS) and plated on 0.6% noble agar-coated plates at a density of 1 × 10^5^ cells/35 mm dish and grown for seven days. For cancer sphere counting, total number of spheres per 20 fields was counted under a 10× phase contrast microscope. For immunoblotting experiments, cells were retrieved from methylcellulose by diluting with phosphate-buffered saline (PBS), centrifuged, and then lysed in western lysis buffer.

### Tumor formation

All animal experiments were reviewed and approved by the Institutional Animal Ethics committee of the Indian Institute of Sciences, Bangalore. Five-week-old athymic^nu/nu^ female nude mice were used to undertake subcutaneous injections of 1 × 10^6^ cells in each flank. Six mice were assigned for each experiment. Tumor size was measured every week using digital vernier calipers up to seven weeks. Tumor volume was calculated using the formula 4/3πr^3^.

### Statistical analysis

All statistical analysis was performed using GraphPad Prism 5.0 software (GraphPad, La Jolla, CA, USA). All data are presented as mean ± standard error of the mean (SEM). *P* values <0.05 were considered to be statistically significant. Statistical analysis was done using paired Student’s *t* test; ^***^represents *P* <0.001, ^**^represents *P* <0.01 and ^*^represents *P* <0.05.

## Results

### Mammosphere formation requires AMPK activity

In order to understand the signaling mechanisms that facilitate MS formation by normal HMECs, we first examined the status of the AMPK pathway, which has recently been implicated in anoikis resistance [15],[16], between adherent HMECs and floating MS. To do so, freshly isolated normal HMECs were cultured for a week in serum-free media in regular tissue culture dishes, leading to the generation of ADH cultures (Figure 1A). In parallel, HMECs were cultured for a week in UL attachment plates that prevent attachment (also referred to as suspension culture), leading to the generation of floating MS (Figure 1A; also see Methods). Immunoblot analysis of cells harvested at the end of a week from both the conditions revealed a significant increase in the levels of Thr^172^ phosphorylated, active forms of AMPKα in MS compared to ADH HMECs; levels of total AMPKα remained unaltered (Figure 1B). In addition, MS showed increased phosphorylation of ACC and Raptor, two well-established targets of AMPK [11], compared to adherent HMECs (Figure 1C). Thus, these data revealed increased activation of AMPK in MS compared to ADH HMEC cultures.

We next investigated the significance of AMPK activation in mammosphere formation. To do so, HMECs were cultured in UL attachment plates for a week in the presence of a pharmacological inhibitor of AMPK, Compound C, a potent reversible inhibitor that is competitive with ATP [28]. Compared to treatment with DMSO (vehicle control), treatment with Compound C led to a decrease in the levels of pACC, confirming the efficacy of the inhibitor, as well as impaired MS formation (Figure 1D), suggesting that AMPK activation may be required for MS formation. To further confirm this, we undertook RNA interference (RNAi)-mediated silencing of AMPK expression. HMECs express both α1 and α2 isoforms of AMPK; however, we detected several fold higher expression of α2 compared to α1 (Figure S1A in Additional file 1). Accordingly, we chose to knockdown AMPK α2 expression using the RNAi approach. Transfection of HMECs cultured in UL attachment plates with siRNA oligos targeting AMPK α2 led to an approximately 80% reduction in the protein levels of AMPK α2 compared to control siRNA transfections (Figure 1E). Knockdown of AMPK α2 did not significantly alter the levels of AMPK α1 expression (Figure S1B in Additional file 1). Further, transfection of AMPK α2 siRNA led to an approximately 80% reduction in AMPK activity, as gauged by a reduction in pACC levels (Figure 1E). Importantly, knockdown of AMPK α2 also impaired MS formation (Figure 1E). An independent set of shRNA constructs targeting AMPK α2 also yielded similar results (Figure S1C in Additional file 1). Taken together, these results revealed the requirement of AMPK signaling for MS formation by normal HMECs.

### AMPK activation inhibits apoptosis

**Figure 2:**
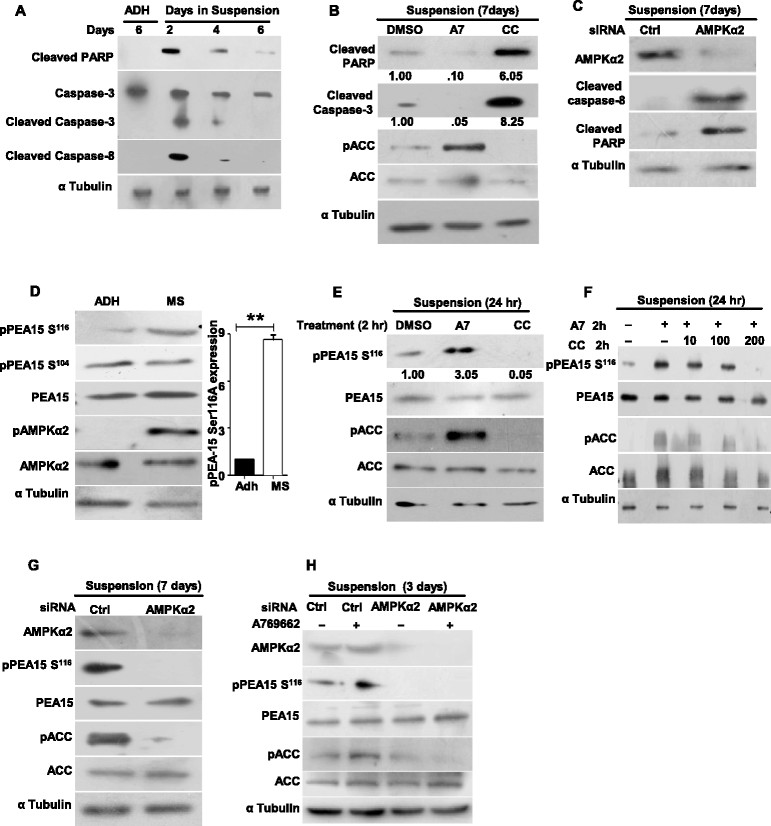
**AMP-activated protein kinase (AMPK) inhibits apoptosis through phosphoprotein enriched in astrocytes 15 kDa (PEA15) phosphorylation.** Primary human mammary epithelial cells (HMECs) cultured under following conditions were harvested and subjected to immunoblot analysis for the specified proteins: **(A)** HMECs seeded in adherent (ADH) condition for six days or in suspension (in ultra-low (UL) plates) for two, four or six days; n = 3. **(B)** HMECs seeded in suspension in the presence of 100 μM AMPK activator (A769662), 10 μM AMPK inhibitor (Compound C) or dimethyl sulphoxide (DMSO) (vehicle control) for a week; n = 3. **(C)** HMECs seeded in UL plates and transfected with control small interfering RNA (siRNA) or siRNA targeting AMPK α2 (Dharmacon) were harvested after one week; n = 4. **(D)** HMECs seeded in ADH condition or in suspension in UL plates for a week. Graph represents densitometric analysis of western blots to quantify phospho PEA15 Ser^116^ relative to total PEA15. Error bars represent standard error of the mean (SEM); (n = 6). **(E)** HMECs seeded in UL plates for 24 hrs and treated with DMSO (vehicle control), 100 μM AMPK activator (A769662), or 10 μM AMPK inhibitor (Compound C) for a period of 2 hrs; n = 3. **(F)** HMECs seeded in UL plates and treated with increasing amounts of AMPK inhibitor Compound C, in the presence of a constant amount of AMPK activator A769662, for 2 hrs; n = 3. **(G)** HMECs seeded in UL plates and transfected with control siRNA or siRNA targeting AMPK α2 and harvested two days following transfection; n = 4. **(H)** HMECs seeded in UL plates and transfected with control siRNA or siRNA targeting AMPK α2 were treated with 100 μM AMPK activator (A769662) after two days following transfection for 2 hrs and harvested, n = 3.

We next investigated how AMPK activation might facilitate the anchorage-independent growth of MS. Detachment of epithelial cells from the matrix triggers ‘anoikis’, a form of apoptosis [3],[4]. Consistent with this, HMECs seeded in UL attachment plates showed an increase in the levels of apoptotic markers (such as cleaved caspase-3, cleaved PARP, and cleaved caspase-8) by day 2 of suspension culture compared to adherent HMECs (Figure 2A). However, from days 2 to 6 in suspension culture, there was a gradual decrease in apoptosis (Figures 2A and Figure S2A in Additional file 2), indicative of anoikis-resistant outgrowth of MS from a subset of HMECs. Further, we noticed an increase in cleaved caspase-8 in HMECs seeded in suspension (Figure 2A), and inhibition of caspase-8 led to an increase in MS formation (Figure S2B in Additional file 2), suggesting the possible involvement of the death receptor pathway in anoikis [29]. To investigate the role of AMPK in MS formation, we assessed the effects of AMPK activation and inhibition on the status of apoptosis in HMECs cultured in UL attachment plates for a week. Compared to vehicle control DMSO, treatment with an AMPK activator A769662 [30], which directly activates the heterotrimeric form of AMPK both allosterically and by inhibiting dephosphorylation [31], led to an increase in pACC levels, while treatment with the AMPK inhibitor Compound C led to a decrease in pACC levels, thus confirming the efficacy of the pharmacological agents (Figure 2B). Further, treatment with A769662 led to a reduction in the apoptotic readouts compared to DMSO vehicle treatment (Figure 2B). This is also consistent with the observed increase in the number of MS generated upon A769662 treatment (data not shown). On the other hand, treatment with Compound C led to an increase in the apoptotic readouts (Figure 2B), which was also consistent with the observed impairment of MS formation (Figure 1D). Further, transfection of HMECs seeded in UL attachment plates with AMPK siRNA, which was previously shown to impair MS formation (Figure 1E), also revealed an increase in the apoptotic readouts (Figure 2C). Together, these data suggested that AMPK activation in suspension might facilitate MS formation by inhibiting matrix deprivation-induced apoptosis.

### AMPK activation leads to increased PEA15 Ser^116^phosphorylation

We next investigated the mechanisms downstream of AMPK activation that might be involved in the inhibition of apoptosis in matrix-deprived HMECs. Phosphorylation of PEA15 at Ser^116^ residue has been shown to block apoptosis by associating with FADD and interfering with the assembly of the death-inducing signaling complex (DISC) [32]. Since this phosphorylation has recently been shown to inhibit apoptosis under the stress of glucose deprivation [23], we investigated the status of PEA15 phosphorylation in HMECs subjected to matrix deprivation using an antibody that specifically recognizes this phosphorylation. Compared to ADH HMECs, MS revealed an increase in Ser^116^ phosphorylation of PEA15 in MS concomitant with AMPK activation (as gauged by pAMPK Thr^172^ levels) (Figure 2D). Since only the double phosphorylated form of PEA15 (at residues Ser^104 and 116^) is associated with anti-apoptotic functions, we investigated the status of Ser^104^ phosphorylation in HMECs. Although Ser^104^ was phosphorylated in MS, there was no significant difference in its levels between ADH HMECs and MS (Figure 2D). The total PEA15 under these two conditions remained unchanged (Figure 2D). Thus, these data revealed an increase in Ser^116^ phosphorylation of PEA15 in MS compared to ADH HMECs.

To investigate the role of AMPK in this process, we investigated the effects of AMPK activation and inhibition on the status of PEA15 Ser^116^ phosphorylation. To do so, we treated HMECs seeded in UL attachment plates with pharmacological activator or inhibitor of AMPK and measured the levels of pPEA15 Ser^116^ by immunoblotting. Compared to DMSO (vehicle)-treated cells, while activation of AMPK with A769662, as measured by an increase in pACC levels, led to an increase in pPEA15 Ser^116^ levels, inhibition of AMPK by Compound C, as measured by a decrease in the levels of pACC, led to a decrease in pPEA15 Ser^116^ levels in these cells (Figure 2E), suggesting the involvement of AMPK upstream of PEA15 Ser^116^ phosphorylation. Moreover, when HMECs seeded in UL attachment plates were treated with increasing concentrations of Compound C in the presence of a constant amount of A769662, we observed a Compound C dose-dependent decrease in the levels of pPEA15 Ser^116^ in parallel with that of pACC (Figure 2F), indicating the specificity of PEA15 Ser^116^ phosphorylation to AMPK activation.

To further confirm the involvement of AMPK upstream of PEA15 phosphorylation, we assessed the effects of AMPK knockdown. Transfection of AMPK α2 siRNA into HMECs seeded in UL attachment plates led to a reduction in the levels of pPEA15 Ser^116^ and pACC compared to control siRNA transfections (Figure 2G). Additionally, in the presence of AMPK α2 siRNA, pharmacological activation of AMPK with A769662 failed to restore PEA15 Ser^116^ phosphorylation (Figure 2H), further supporting an AMPK-dependent phosphorylation of PEA15 Ser^116^ in MS. Furthermore, inhibition of Akt and CaMKII, two known upstream kinases of PEA15 [33], failed to affect PEA15 Ser^116^ phosphorylation in suspension (Figure S2 C-E in Additional file 2). In addition, co-IP experiments revealed increased association of pPEA15 Ser^116^ with FADD upon AMPK activation, while its inhibition reduced this association (Figure S2F in Additional file 2). Taken together, these data suggested a role for Ser^116^ phosphorylated PEA15 downstream of AMPK activation in the inhibition of apoptosis during MS formation.

### PEA15 and its phosphorylation at Ser^116^are critical for mammosphere formation

**Figure 3:**
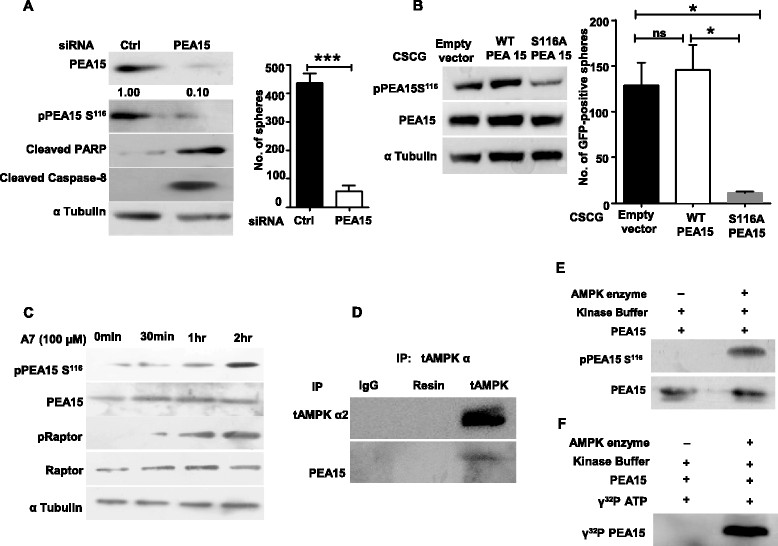
**Phosphoprotein enriched in astrocytes 15 kDa (PEA15) is a novel AMP-activated protein kinase (AMPK) substrate. (A)** Primary human mammary epithelial cells (HMECs) cultured in ultra-low (UL) plates were transfected with control small interfering RNA (siRNA) or siRNA targeting PEA15. Two days posttransfection, a quarter of the cells were harvested and subjected to immunoblot analysis for the specified proteins (i), while the rest were scored for total number of mammospheres (MS) formed at the end of a week (ii). Error bars represent standard error of the mean (SEM); n = 4. **(B)** Primary HMECs cultured in UL plates were infected with control (empty vector) CSCG lentivirus, or those encoding for PEA15-WT or PEA15-S116A mutant with similar infection efficiencies (as scored by GFP-positive cells). Two days following infection, 1 × 10^5^ transduced cells were seeded in methylcellulose and total number of GFP-positive mammospheres formed was counted at the end of a week. Error bars represent standard error of the mean (SEM); n = 3. **(C)** Primary HMECs seeded in UL plates were treated with AMPK activator A769962 (100 μM) for 0 min, 30 min, 1 hr and 2 hrs, and subjected to immunoblot analysis for the specified proteins; n = 3. **(D)** Primary HMECs seeded in UL plates were subjected to immunoprecipitation with total AMPK antibody followed by immunoblotting for specified proteins. Rabbit immunoglobulin G (IgG)-treated lysate and resin alone served as controls, n = 3. **(E)** Commercially procured heterotrimeric AMPK and pure PEA15 were incubated in an *in vitro* kinase assay buffer in the presence of 50 μM *adenosine triphosphate* (ATP) and 0.1 mM AMP for 30 min, and then subjected to immunoblot analyses with commercially available antibodies that specifically recognize PEA15 Ser^116^ phosphorylation; total PEA15 antibody served as a loading control. **(F)** Autoradiography of *in vitro* kinase assay using pure AMPK and PEA15 proteins performed in the presence of γ^32^P-labeled ATP (0.3 μCi) for 30 min; n = 3.

To understand the significance of PEA15 and its phosphorylation in the inhibition of apoptosis and MS formation, we investigated the effects of PEA15 knockdown. Transfection of HMECs cultured in UL attachment plates with PEA15 siRNA led to approximately 90% decrease in the levels of total PEA15 in these cells (Figure 3A). Consistent with this, these cells also showed reduced levels of pPEA15 Ser^116^ (Figure 3A). Further, transfection with PEA15 siRNA led to an increase in apoptosis, as detected by increased levels of cleaved PARP and cleaved caspase-8 (Figure 3A), and also impaired MS formation (Figure 3A), together revealing the requirement of PEA15 in MS formation.

We next investigated the role of Ser^116^ phosphorylation of PEA15 in MS formation. To do so, we undertook lentiviral-mediated expression of the S116A nonphosphorylatable mutant of PEA15 in HMECs cultured in UL attachment plates. Compared to empty vector and WT-PEA15-transduced HMECs, those transduced with PEA15 S116A mutant had reduced levels of pPEA15 Ser^116^, and were also impaired in their MS-forming potential (Figure 3B). Thus, these data revealed the importance of PEA15 and its phosphorylation on Ser^116^ for MS formation.

### AMPK directly phosphorylates PEA15

Since our data revealed an AMPK-dependent phosphorylation of PEA15 in MS, we next investigated if PEA15 could be a direct substrate for AMPK. The kinetics of PEA15 Ser^116^ phosphorylation following AMPK activation paralleled that of two well-established substrates of AMPK, ACC (Figure 2F) and Raptor [34] (Figure 3C), suggesting that PEA15 could be a direct target for AMPK. Clustal W alignment revealed that four out of eight residues flanking Ser^116^ in PEA15 (Figure S3A in Additional file 3) showed a significant match with the AMPK consensus motif [34],[35]. We also found that the PEA15 Ser^116^ flanking sequences are highly conserved among mammals and it matches with the already characterized AMPK phosphorylation sites in various substrates (Figure S3B and C in Additional file 3). These analyses suggested that PEA15 could be a substrate for AMPK. To assess this, we first gauged if the two proteins interacted. Co-IP assays undertaken with MS lysates and MCF7 cells expressing myc-tagged AMPK revealed association of AMPK with PEA15 (Figure 3D and Figure S3D in Additional file 3). We then assessed if AMPK can directly phosphorylate PEA15. To do so, we undertook *in vitro* kinase assays with commercially procured PEA15 and active heterotrimeric form of AMPK proteins. Subsequent immunoblot analysis using an antibody that specifically recognizes PEA15 phosphorylated at Ser^116^ residue revealed direct phosphorylation of this residue in the presence of active AMPK (Figure 3E). In addition, *in vitro* kinase assays performed in the presence of [γ^32^P] ATP also confirmed direct phosphorylation of PEA15 by AMPK (Figure 3F). Thus, these results revealed that PEA15 could indeed be a novel target of AMPK.

Taken together, these data revealed that activation of AMPK and phosphorylation of PEA15 at Ser^116^ facilitates MS formation by inhibiting apoptosis. Our study thus identifies a novel AMPK-PEA15 axis in the anoikis-resistant outgrowth of a subset of normal HMECs as MS.

### AMPK-PEA15 axis is critical for the anchorage-independent growth of breast cancer cells

**Figure 4:**
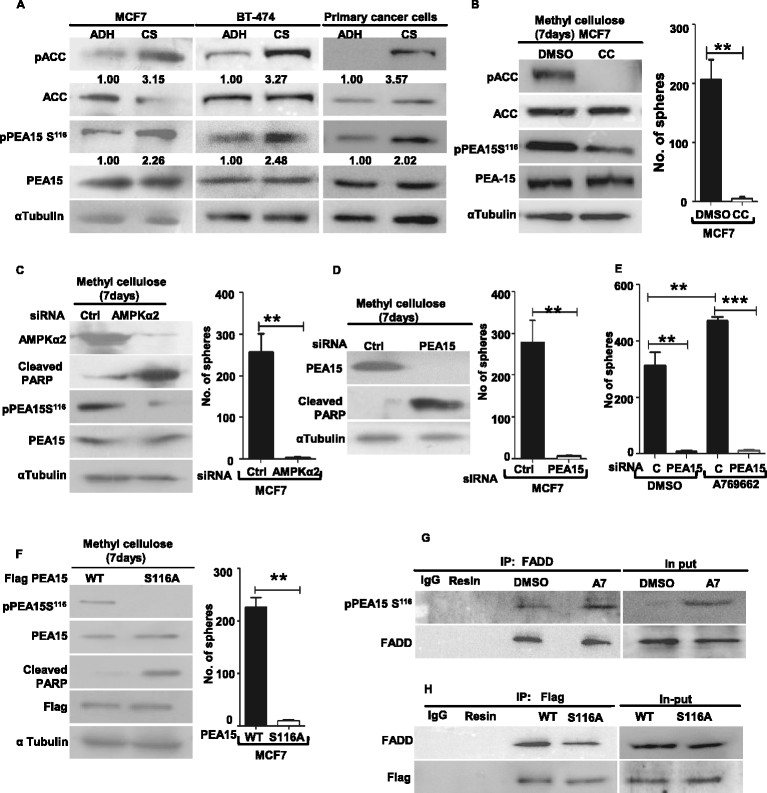
**AMP-activated protein kinase (AMPK)-phosphoprotein enriched in astrocytes 15 kDa (PEA15) axis is critical for the anchorage-independent growth of breast cancer cells. (A)** Michigan Cancer Foundation 7 breast cancer cell line (MCF7), BT-474, and primary breast cancer-derived cells cultured in adherent (ADH) condition or as cancer spheres (CS) for a week were harvested and subjected to immunoblotting; n = 3. **(B)** MCF7 cells cultured in methylcellulose for a week in the presence of 10 μM AMPK inhibitor Compound C or dimethyl sulphoxide (DMSO) (vehicle control) were subjected to immunoblotting. Graph represents number of spheres formed/20 fields. Error bars represent standard error of the mean (SEM); n = 3. **(C-F)** Adherent MCF7 breast cancer cells were transfected with specified small interfering RNA (siRNA) oligos/plasmids (see below). Two days posttransfection, 1 × 10^5^ cells/35 mm dish were seeded in methylcellulose. After 48 hrs, cells were retrieved from some dishes and subjected to immunoblot analyses for the specified proteins. Parallel dishes were allowed to form spheres; graph represents sphere formation at the end of a week, error bar represents SEM: **(C)** control siRNA or siRNA targeting AMPK α2, n = 4; **(D)** control siRNA or siRNA targeting PEA15, (n = 4); **(E)** control siRNA or siRNA targeting PEA15 and seeded in methylcellulose in the presence of 100 μM AMPK activator, A769662; DMSO served as vehicle control, n = 4; **(F)** transfected with CSCG constructs expressing flag-tagged wild-type (WT) or S116A mutant of PEA15, n = 4. **(G)** MDAMB231 cells were treated with DMSO or 100 μM AMPK activator, A769662, and immunoprecipitated with anti-Fas-associated death domain protein (FADD) antibody. The immunoprecipates were resolved by SDS-PAGE followed by immunoblotting for specified proteins, n = 3. **(H)** BT 474 cells stably expressing flag-tagged WT-PEA15 or PEA15-S116A mutant were seeded in methyl cellulose for two days, retrieved and immunoprecipitated with anti-flag antibody. The immunoprecipitates were resolved by SDS-PAGE followed by immunoblotting for specified proteins, n = 3.

Since anchorage-independent growth is a fundamental property of solid tumors, we next investigated if the AMPK-PEA15 axis played any role in the tumorigenicity of breast cancer cells. For this, we first compared the status of AMPK and PEA15 signaling in MCF7 and BT474 breast cancer cell lines grown as ADH monolayer cultures or as cancer spheres in methylcellulose (Figure S4A in Additional file 4; also see Methods). Immunoblot analysis revealed a significant increase in the levels of pACC in cancer spheres compared to ADH cultures (Figure 4A), indicating activation of AMPK pathway in matrix-deprived cancer cells, consistent with recent reports [15],[17]. Compared to ADH cultures, cells growing as cancer spheres also showed an increase in the levels of pPEA15 Ser^116^ (Figure 4A); levels of total PEA15 did not show any significant difference. Primary breast cancer-derived cells also showed elevated AMPK activity and pPEA15 Ser^116^ levels compared to those growing in ADH condition (Figure 4A). Thus, similar to HMECs, breast cancer cells also showed an increase in AMPK activity and PEA15 Ser^116^ phosphorylation upon matrix deprivation (Figure S4B in Additional file 4), suggesting the possible involvement of an AMPK-PEA15 axis in the survival of matrix-deprived breast cancer cells too.

We next investigated the requirement of AMPK in the anchorage-independent growth of breast cancer cells as spheres. Treatment with AMPK inhibitor Compound C (Figures 4B, and Figure S4C and D in Additional file 4) or knockdown of AMPK α2 (Figures 4C and Figure S4 E-G in Additional file 4) dramatically decreased sphere formation in MCF7, MDAMB231 and BT 474 breast cancer cell lines. Further, inhibition or knockdown of AMPK also impaired PEA15 Ser^116^ phosphorylation in these cells (Figure 4B and C, Figure S4 C-E in Additional file 4), and increased apoptosis, as revealed by increased PARP levels (Figure 4C and Figure S4E in Additional file 4). Thus, these data revealed the requirement of AMPK activation for sphere formation by breast cancer cells.

We next assessed the importance of PEA15 and its phosphorylation downstream of AMPK activation in the anoikis-resistant growth of breast cancer cells. Compared to control siRNA, transfection with PEA15 siRNA led to reduced sphere formation (Figure 4D), which could not be rescued by pharmacological activation of AMPK (Figure 4E). Furthermore, overexpression of the nonphosphorylatable S116A mutant of PEA15 also dramatically decreased sphere formation in all the three cell lines compared to WT PEA15 (Figure 4F, and Figure S4 H and I in Additional file 4). In addition, overexpression of PEA15 S116A mutant led to increased apoptosis, as detected by cleaved PARP levels (Figure 4F), which was also not rescued by pharmacological activation of AMPK (data not shown). Thus, these set of experiments highlighted the importance of PEA15 and its phosphorylation at Ser^116^ in breast cancer sphere formation.

We further gauged the importance of AMPK activation and PEA15 Ser^116^ phosphorylation in its association with FADD in breast cancer cells. We observed increased association of pPEA15 Ser^116^ with FADD under conditions of AMPK activation (Figure 4G). Also, compared to WT PEA15-expressing cells, those expressing the PEA15 S116A mutant showed less association of PEA15 with FADD (Figure 4H) highlighting the importance of Ser^116^ phosphorylation of PEA15 for its association with FADD in these cells, in keeping with previous reports [22],[36]. Together, these experiments revealed the importance of the AMPK-PEA15 axis in the inhibition of apoptosis during the anchorage-independent growth of breast cancer cells *in vitro*.

**Figure 5:**
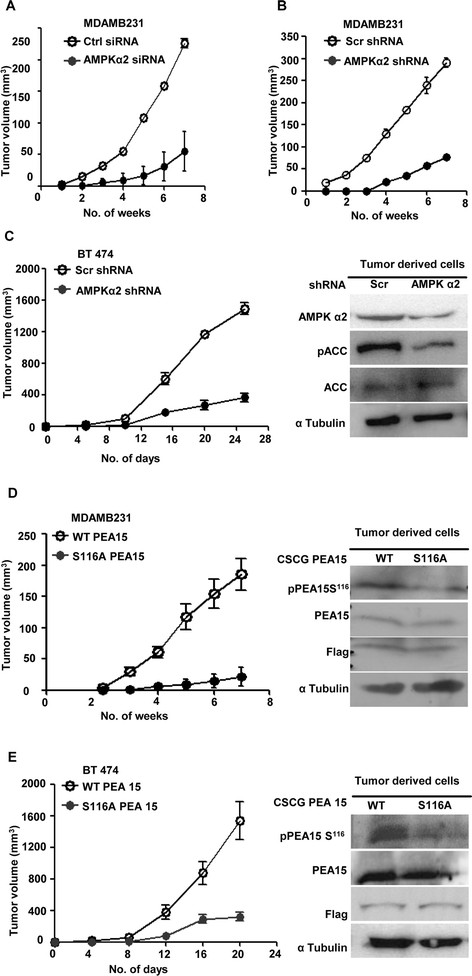
**AMP-activated protein kinase (AMPK)-phosphoprotein enriched in astrocytes 15 kDa (PEA15) axis is critical for tumorigenicity of breast cancer cells. (A)** Breast cancer cell line derived from metastatic site (pleural effusion) (MDAMB231) cells were transfected with control small interfering RNA (siRNA) or siRNA targeting AMPK α2 and injected subcutaneously (1 × 10^6^ cells/injection) into six nude mice (control siRNA transfected cells in the left flank, and AMPK α2 siRNA-transfected cells in the right flank) and monitored for tumor formation for seven weeks. (**B** and **C**) MDAMB231 cells (B) and BT 474 cells (C) stably expressing short hairpin RNA (shRNA) pool against AMPK α2 or scrambled shRNA were introduced subcutaneously (1 × 10^6^ cells/injection) into three nude mice and tumor formation was monitored for the indicated time period. Control shRNA-expressing cells were injected in the left flank, and AMPK α2 shRNA-expressing cells in the right flank. Tumors were resected and tumor-derived cells subjected to immunoblotting for specified proteins. (**D** and **E**) MDAMB231 cells **(D)** and BT 474 cells **(E)** stably expressing CSCG-WT-Flag PEA15 or CSCG-S116A-Flag-PEA15 were injected subcutaneously (1 × 10^6^ cells/injection) into six nude mice and tumor formation was monitored for the indicated time period. Cells stably expressing wild-type (WT)-PEA15 were injected in the left flank, while cells stably expressing S116A-PEA15 were injected in the right flank. Tumors were resected and tumor-derived cells subjected to immunoblotting for specified proteins.

We next investigated the relevance of the AMPK-PEA15 signaling axis in the tumorigenicity of breast cancer cells *in vivo*. To do so, MDAMB231 cells transiently transfected with control or AMPK α2 siRNA were injected subcutaneously into either flanks of immunocompromised mice. Knockdown of AMPK (Figure S5A in Additional file 5) led to impairment of tumor formation compared to control siRNA transfections (Figure 5A). Tumor formation was also impaired in MDAMB231 and BT 474 cells stably expressing shRNA constructs targeting AMPK α2 (Figure 5B and C). Further, MDAMB231 and BT 474 cells stably expressing the nonphosphorylatable mutant of PEA15 were also markedly impaired in their tumor-forming potential compared to WT PEA15-expressing cells (Figure 5C and D), together suggesting that AMPK activation and PEA15 phosphorylation are both necessary for tumor formation by breast cancer cells.

Thus, our study uncovers a hitherto unknown AMPK-PEA15 signaling axis in the context of anoikis-resistant growth of normal and cancerous mammary epithelial cells, suggesting that targeting this axis could serve as a novel strategy to curb cancer cell dissemination and metastasis.

## Discussion

Anoikis is a physiologically relevant process, which plays an important role in normal development as well as in the maintenance of tissue homeostasis throughout adult life. By preventing detached epithelial cells from colonizing elsewhere, anoikis serves as a barrier to cancer progression. Thus, acquisition of anoikis resistance is considered to be a prerequisite for solid tumor metastasis. Hitherto considered a fundamental property of cancer cells, however, recent studies have intriguingly identified the anoikis-resistant outgrowth of a small subset of normal epithelial cells as floating spheroids *in vitro* in multiple epithelial systems including the mammary, prostate, and salivary gland [7],[37],[38]. Thus, it is conceivable that epithelial cancer cells exploit mechanisms already inherent within their normal counterparts for survival in circulation. In this study, we have identified a novel AMPK-PEA15 axis that confers survival signals to facilitate the anchorage-independent growth of normal HMECs as MS. We further show that the same axis plays a critical role in the anchorage-independent growth of breast cancer cells both *in vitro* and *in vivo*.

Though our study has identified a role for AMPK in the anchorage-independent growth of normal HMECs as mammospheres *in vitro*, the *in vivo* relevance of this phenomenon in mammary gland development and homeostasis remains unclear at this point of time. Mammary cells, like most epithelial cells, grow attached to the basement membrane *in vivo. In vitro*, adhesion promotes differentiation, while the nonadherent MS are enriched in stem/progenitor cells [7]. Such undifferentiated cells *in vivo* may reside in niches lacking proper ECM attachment, and may employ survival signals emanated by AMPK activation. Furthermore, during post-lactational involution, mammary cells undergo massive apoptosis thought to be triggered by the degradation of the ECM [39]. Regrowth of the mammary gland during the next pregnancy is believed to be due to leftover stem/progenitor-like cells [40] that may utilize AMPK signaling for their survival. However, no mammary phenotype has so far been reported in mice lacking AMPK α 1 or 2; double knockout (KO) mice are embryonically lethal [37]. Perhaps, the presence of one isoform may have compensated for the other since mammary cells express both the AMPK α isoforms. Thus, the generation of conditional, mammary gland-specific AMPK α 1 + 2 KO mice might reveal important information on the role of AMPK in the development and maintenance of the adult mammary gland. Further, the presence of a small population of cells with the capability of anoikis-resistant growth within the normal mammary gland implies that these cells could be targets for oncogenic events leading to luminal filling and breast carcinogenesis. Indeed, we recently showed that the tumorigenic transformation of MS-derived cells led to the generation of invasive ductal carcinoma harboring cancer stem-like cells [27].

The role of AMPK in the context of cancer has remained controversial, with earlier studies implicating a tumor-suppressive role for the LKB1-AMPK axis, while recent studies unfurling tumor-promoting roles [41]. For example, the AMPK upstream kinase LKB1 is a tumor suppressor that is mutated in Peutz-Jegher syndrome [11]. Additionally, AMPK has been shown to regulate the activity of p53 and pRb leading to the inhibition of cancer cell growth [42],[43]. In contrast, AMPK has been demonstrated to trigger adaptive responses in cancer cells under metabolically depressed states like hypoxia and low glucose conditions [14],[44]. Additionally, AMPK has been shown to confer resistance to human glioma and mouse fibrosarcoma cells upon cisplatin treatment [45]. Indeed, several kinds of stresses associated with tumor progression, including hypoxia, nutrient deprivation and reactive oxygen species, are known triggers that activate AMPK. In this study, we show that the stress of anchorage-deprivation leads to AMPK activation and this is required for the anchorage-independent growth of breast cancer cells. Our data is consistent with two recent studies that have associated AMPK with matrix detachment [17],[46]. Of note, when matrix-deprived cancer cells reattach, AMPK activity gradually decreases to basal level (data not shown). Thus, AMPK activation might serve as a reversible switch in cancer cell dissemination and metastasis.

Upon activation, AMPK brings about energy homeostasis by inhibiting anabolic pathways and activating catabolic pathways [47],[48]. In line with this, one study showed the maintenance of NADPH homeostasis as a predominant mechanism by which AMPK enables cancer cell survival during stress conditions and promotes tumor formation [17]. In another study, AMPK-mediated mTORC1 inhibition and suppression of protein synthesis as a means for bioenergetic conservation was implicated in anoikis resistance [15]. In our study, we show that AMPK activation leads to Ser^116^ phosphorylation of PEA15 that promotes its association with FADD, thereby protecting matrix-deprived cells from apoptosis. Thus, pleiotropic effects downstream of AMPK activation are likely to contribute to anoikis resistance. Earlier studies had implicated a role for PI3K/Akt, epidermal growth factor receptor (EGFR) activation, and Bcl-2 in the survival of cancer cells under detachment [49]-[51]. Thus, stress signaling pathways likes AMPK might function in concert with these other signaling pathways to enable anchorage-independent survival of cancer cells.

Signaling by the death-receptor proteins, such as Fas and TNF-R1, leads to the formation of the death-induced silencing complex (DISC) consisting of death-receptor, FADD, and caspase-8 which initiates the caspase cascade [32]. Death receptor-mediated signaling has been implicated in anoikis [9],[29]. Matrix detachment leads to overexpression of Fas L and Fas R, while overexpression of dominant-negative form of FADD inhibits anoikis [9],[29]. Further, matrix attachment has been shown to protect cells from Fas-induced apoptosis, whereas matrix detachment sensitizes cells to Fas-mediated apoptosis [9]. Our data revealed increased caspase-8 cleavage in matrix-detached HMECs, while inhibition of caspase-8 led to increase in MS formation. However, how matrix detachment leads to the activation of the death-receptor pathway in these cells needs to be further explored.

**Figure 6:**
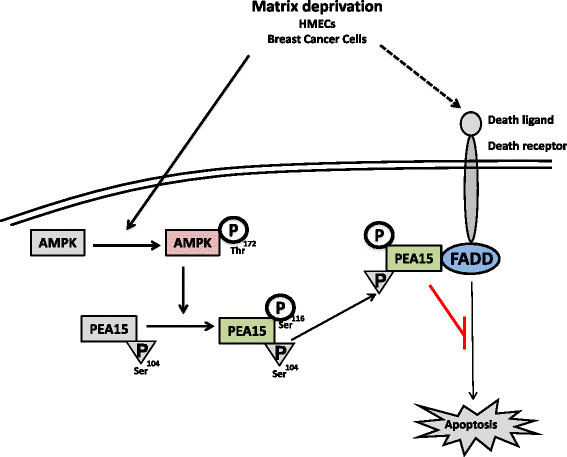
**AMP-activated protein kinase (AMPK) activation inhibits anoikis through phosphoprotein enriched in astrocytes 15 kDa (PEA15) Ser**^**116**^**phosphorylation.** Matrix deprivation triggers apoptosis, at least in part, through the death-receptor pathway. We show that in human mammary epithelial cells (HMECs) and breast cancer cells growing in suspension, matrix deprivation leads to increased phosphorylation and activation of AMPK that in turn phosphorylates PEA15 at Ser^116^, thus promoting its association with Fas-associated death domain protein (FADD) and inhibition of apoptosis.

The PEA15 protein associates with ERK in the cytoplasm, restricting its nuclear entry, thus functioning as a tumor suppressor. However, phosphorylation of PEA15 leads to changes in its binding partners, and converts it into a tumor promoter [21]. Phosphorylation at the Ser^116^ position enables its association with FADD, leading to the disruption of the DISC assembly and blocking apoptosis [21]. Both Akt and CaMKII have previously been identified as upstream kinases for PEA15 Ser^116^ phosphorylation. However, our study revealed that under matrix-deprived condition, inhibition of Akt or CaMKII failed to alter the levels of PEA15 Ser^116^ phosphorylation in HMECs. In contrast, inhibition or knockdown of AMPK impaired this phosphorylation. Our data further revealed direct phosphorylation of PEA15 Ser^116^ by AMPK, thus identifying AMPK as an upstream kinase of PEA15. It is plausible that multiple stress kinases employ similar downstream mechanisms to mediate their functions, and perhaps do so in a context-dependent manner. Under the stress of matrix deprivation, our data revealed that AMPK plays a predominant role in the phosphorylation of PEA15. Thus, matrix detachment may trigger anoikis, at least in part, through the death-receptor pathway; our data shows that AMPK activation upon matrix deprivation helps overcome anoikis by phosphorylating PEA15 at Ser^116^ position, which associates with FADD and blocks apoptosis (Figure 6). Further, PEA15 phosphorylation at Ser^116^ was shown to be required for blocking apoptosis in glucose-deprived glioblastoma cells [2]. Since glucose deprivation is known to activate AMPK [14],[52], it is possible that AMPK may be involved with PEA15 phosphorylation under the stress of glucose deprivation too. Of note, both AMPK and its consensus motif on PEA15 are conserved across species. Thus, AMPK-mediated phosphorylation of PEA15 could be an evolutionarily conserved pro-survival mechanism to withstand multiple stress conditions.

## Conclusions

Understanding the molecular mechanisms that contribute to anoikis resistance is important for designing strategies to prevent cancer cell dissemination and spread. In this study, we have uncovered a novel AMPK-PEA15 signaling axis that contributes to the anoikis-resistant growth of normal HMECs as MS. We further show that this signaling axis is also critical for the anchorage-independent growth of breast cancer cells both *in vitro* and *in vivo*, suggesting that cancerous breast cells may exploit a mechanism pre-existent within their normal counterparts to their benefit. Thus, our study reveals targeting the AMPK-PEA15 axis as a novel therapeutic approach in the prevention of breast cancer dissemination and metastasis.

## Additional files

## Electronic supplementary material

Additional file 1: Supplementary Figure S1.(PDF 316 KB)

Additional file 2: Supplementary Figure S2.(PDF 358 KB)

Additional file 3: Supplementary Figure S3.(PDF 201 KB)

Additional file 4: Supplementary Figure S4.(PDF 534 KB)

Additional file 5: Supplementary Figure S5.(PDF 127 KB)

Below are the links to the authors’ original submitted files for images.

Authors’ original file for figure 1

Authors’ original file for figure 2

Authors’ original file for figure 3

Authors’ original file for figure 4

Authors’ original file for figure 5

Authors’ original file for figure 6

## Abbreviations

ACC: acetyl-Co-A carboxylase
ADH: adherent
AMPK: AMP-activated protein kinase
ATP: *adenosine triphosphate*
CaMKII: calcium/calmodulin-dependent protein kinase II
DISC: death-induced silencing complex
DMEM: Dulbecco’s modified Eagle’s medium
DMSO: dimethyl sulphoxide
ECM: extracellular matrix
EGFR: epidermal growth factor receptor
ERK: extracellular signal-regulated kinase
FADD: Fas-associated death domain protein
FBS: fetal bovine serum
HMECs: human mammary epithelial cells
IP: immunoprecipitation
LKB1: liver kinase B1
MCF7: Michigan Cancer Foundation 7 breast cancer cell line
MDAMB231: breast cancer cell line derived from metastatic site (pleural effusion)
MS: mammospheres
PARP: poly(ADP-ribose) polymerase
PEA15/PED: phosphoprotein enriched in astrocytes 15 kDa/phosphoprotein enriched in diabetes
RNAi: RNA interference
siRNA: small interfering RNA
shRNA: short hairpin RNA
UL: ultra-low
WT: wild type
