# Supplementary material for: Identification of a novel AMPK-PEA15 axis in the anoikis-resistant growth of mammary cells
Source: Breast Cancer Res. 2014 Aug 6;16:420. doi: 10.1186/s13058-014-0420-z (PMC4303232; doi:10.1186/s13058-014-0420-z)
Supplement: Supplementary file 1 — Additional file 1: Supplementary Figure S1.(PDF 316 KB) [file 13058_2014_420_MOESM1_ESM.pdf]

Supplementary Figure S1

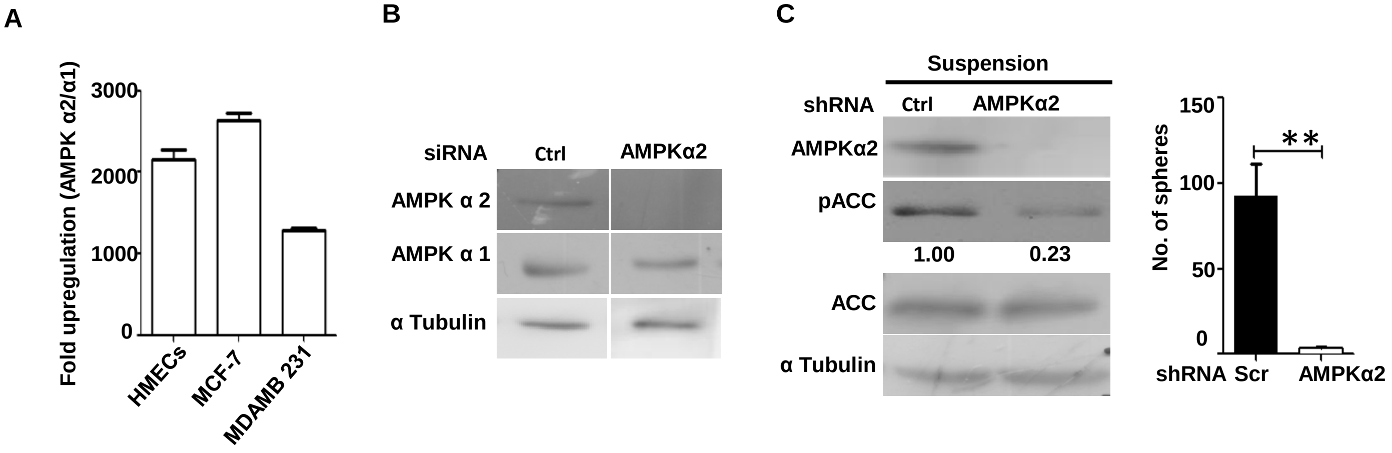

Supplementary Figure S1

A) Bar graph represents fold up-regulation of mRNA expression of AMPK α2 relative to α1 in HMECs and breast cancer cells (MCF7 and MDA MB 231) as assessed by real time qPCR analysis. Total RNA was isolated using TRI reagent. cDNA synthesis was performed using Gene-Amp RNA PCRc DNA synthesis kit. qRT-PCR was carried out using SYBR green master mix in an ABI 7500 RT-PCR system and analyzed using the ΔCt method with β2 microglobulin as internal controls. The primer sequences used are:

PRKAA1 (AMPK alpha1) FW primer: GACAGCCGAGAAGCAGAAAC
PRKAA1 (AMPK alpha1) RV primer: AGGATGCCTGAAAAGCTTGA
PRKAA2 (AMPK alpha2) FW primer: ACCAGCTTGCAGTGGCTTAT
PRKAA2 (AMPK alpha2) RV primer: CAGTGCATCCAATGGACATC
β2M (Beta 2 microglobulin) FW primer: CCTGAATTGCTATGTGTCTGGG
β2M (Beta 2 microglobulin) RV primer: TGATGCTGCTTACATGTCTCGA

B) Primary HMECs seeded in ultra low attachment plates were transfected with control siRNA or siRNA targeting AMPK α2 (Dharmacon). Two days post transfection, a quarter of the cells were harvested and subjected to immunoblot analyses for the specified proteins, n=3.

C) Primary HMECs seeded in ultra-low attachment plates were transduced with retroviruses expressing control shRNA or shRNA targeting AMPK α2 (Origene). Two days following infection, cells were treated with puromycin (0.5 µg/ml) for 48 hrs. Thereafter, cells were trypsinized, a quarter of the cells were harvested for immunoblot analyses for the specified proteins, while $2x10^5$ live cells were seeded in ultra-low attachment plates for a week to score for mammosphere formation. Error bars represent SEM; n=3.
