# Supplementary material for: Identification of a novel AMPK-PEA15 axis in the anoikis-resistant growth of mammary cells
Source: Breast Cancer Res. 2014 Aug 6;16:420. doi: 10.1186/s13058-014-0420-z (PMC4303232; doi:10.1186/s13058-014-0420-z)
Supplement: Supplementary file 2 — Additional file 2: Supplementary Figure S2.(PDF 358 KB) [file 13058_2014_420_MOESM2_ESM.pdf]

Supplementary Figure S2

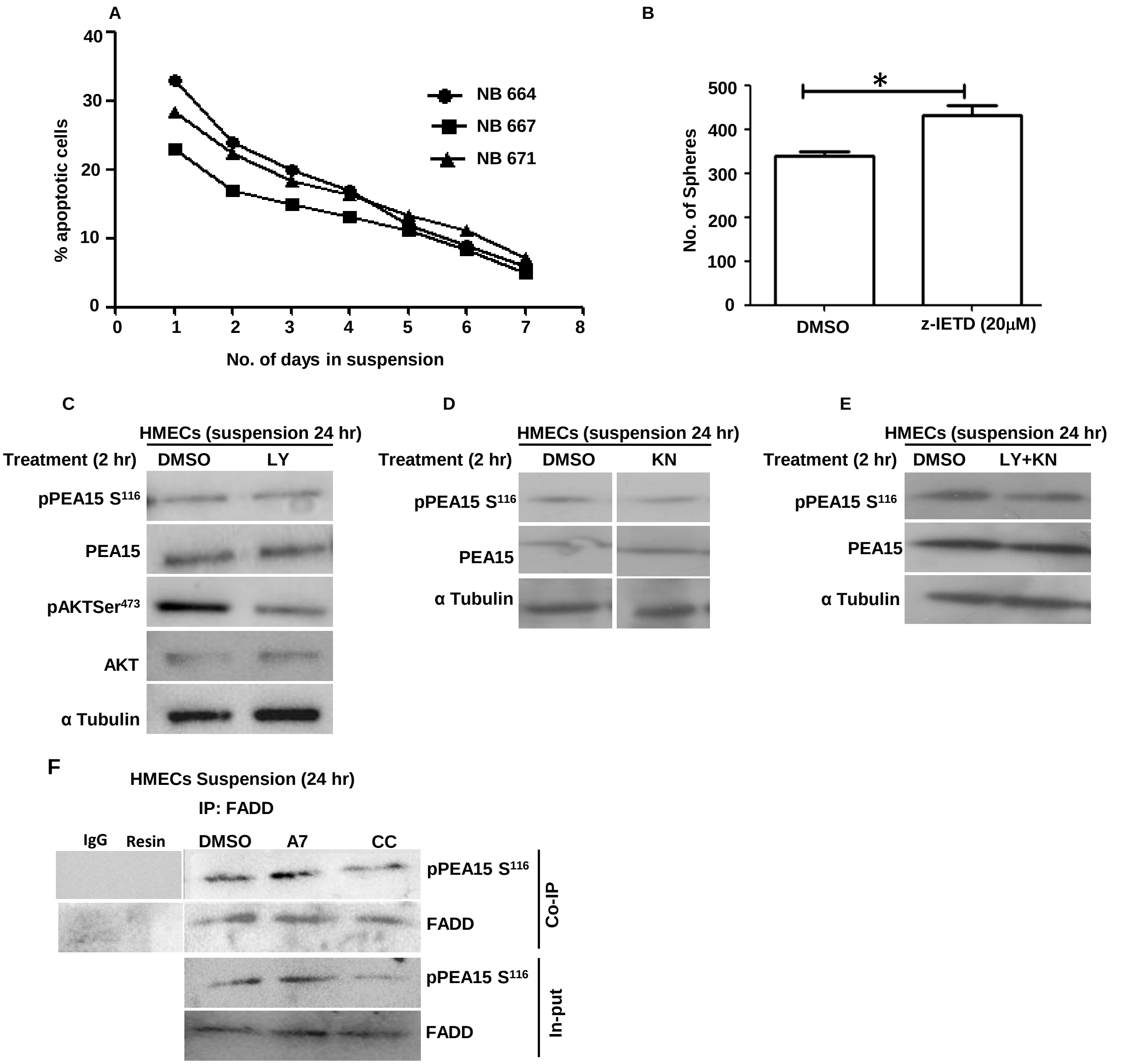

Supplementary Figure S2

A) Primary HMECs seeded in ultra-low attachment plates were harvested every 24 hours for a period of 7 days and stained with Annexin V-FITC and PI and subjected to flow cytometry analyses. Graph shows the % of Annexin V + PI positive apoptotic cells at indicated time points.

B) Primary HMECs were seeded in ultra-low attachment plates for a week in the presence of vehicle control DMSO or caspase inhibitor z-IETD (20 μM). Graph shows the total number of mammospheres formed at the end of a week.

C-E) Primary HMECs seeded in ultra-low attachment plates were treated with DMSO (vehicle control) and 100 μM PI3K inhibitor LY294002 (LY) (C), 50 μM CaMKII inhibitor KN-93 (KN) (D), or LY+KN (E) for two hrs. Cells were harvested after 2 hrs and subjected to immunoblotting for specified proteins; n=3.

F) Primary HMECs cultured in ultra-low attachment plates for were treated with 100 μM AMPK activator (A769662), 10 μM AMPK inhibitor (Compound C), or DMSO (vehicle control) for 24 hrs and subjected to immunoprecipitation with anti-FADD antibody. The immunoprecipitates were resolved by SDS-PAGE and the western blot probed with specified antibodies; n=3.
