# Supplementary material for: Identification of a novel AMPK-PEA15 axis in the anoikis-resistant growth of mammary cells
Source: Breast Cancer Res. 2014 Aug 6;16:420. doi: 10.1186/s13058-014-0420-z (PMC4303232; doi:10.1186/s13058-014-0420-z)
Supplement: Supplementary file 3 — Additional file 3: Supplementary Figure S3.(PDF 201 KB) [file 13058_2014_420_MOESM3_ESM.pdf]

Supplementary Figure: S3

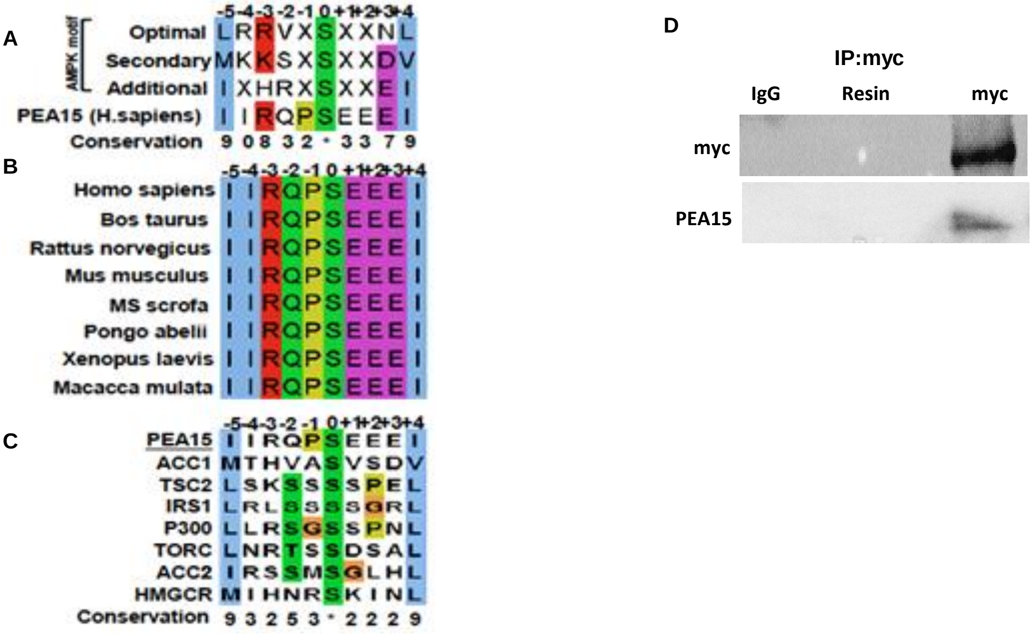

Supplementary Figure S3

A-C) Clustal W analysis comparing the published AMPK motifs [35] to the regions flanking (-5 to +4) of PEA15 Ser^{116} residue (A), comparing PEA15 Ser^{116} flanking regions (-5 to +4) among different mammals (B), and comparing PEA15 Ser^{116} flanking regions (-5 to +4) to AMPK phosphorylation sites in known AMPK targets (C).

D) MCF7 cells transfected with myc-AMPK construct were subjected to immunoprecipitation with anti-myc antibody. The immunoprecipitates were resolved by SDS-PAGE and the western blot was probed with antibodies against total-AMPK.
