# Supplementary material for: Identification of a novel AMPK-PEA15 axis in the anoikis-resistant growth of mammary cells
Source: Breast Cancer Res. 2014 Aug 6;16:420. doi: 10.1186/s13058-014-0420-z (PMC4303232; doi:10.1186/s13058-014-0420-z)
Supplement: Supplementary file 4 — Additional file 4: Supplementary Figure S4.(PDF 534 KB) [file 13058_2014_420_MOESM4_ESM.pdf]

Supplementary Figure S4

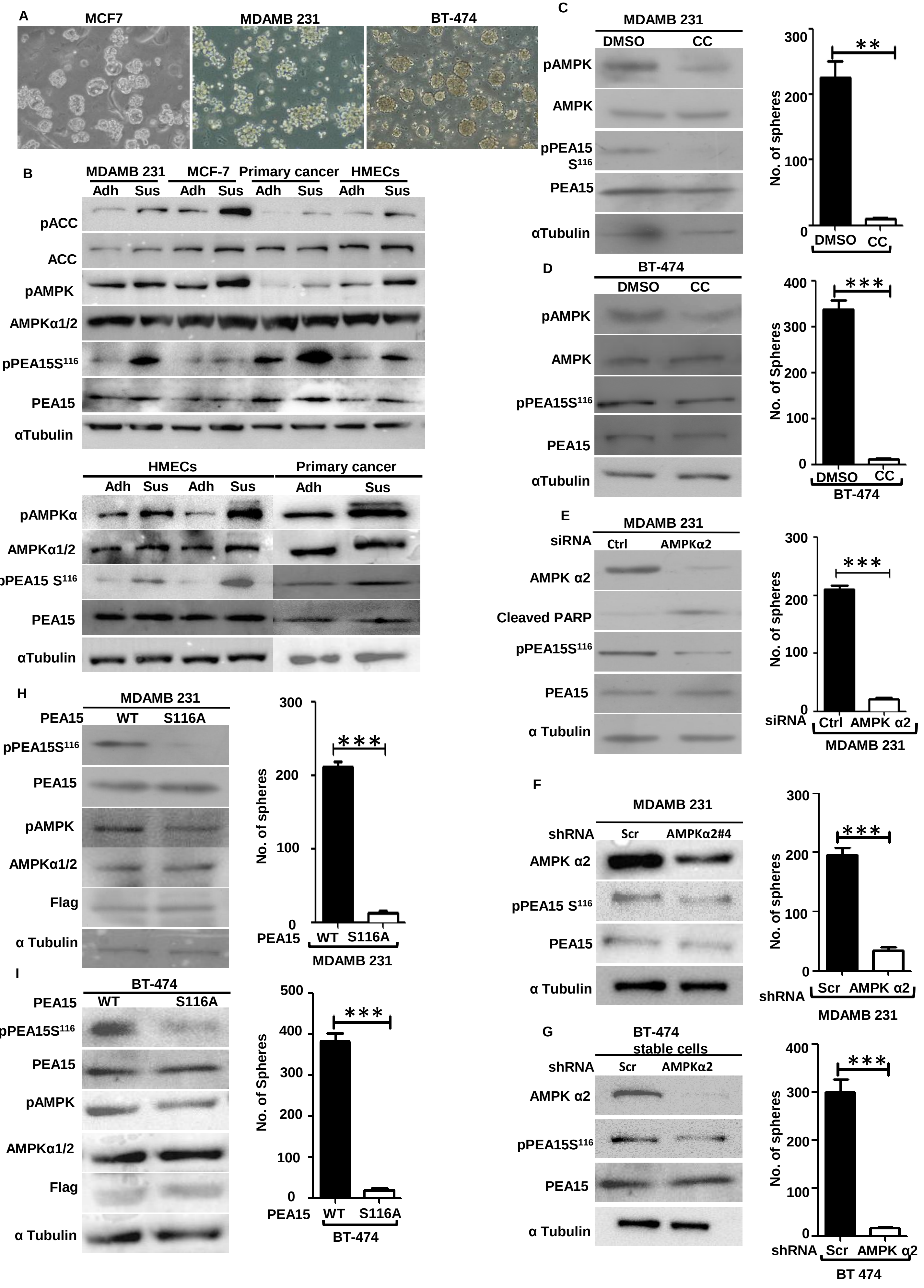

Supplementary Figure S4

A) Phase contrast images represent MCF7, MDAMB231 and BT 474 cells cultured in adherent (ADH) condition or in methylcellulose as cancer spheres (CS) for a week.

B) Immunoblot analyses of cancer cell lines and multiple samples of primary HMECs and primary breast cancer-derived cells grown as adherent culture or in suspension.

C-F): ***Effect of AMPK inhibition or knockdown on sphere formation by breast cancer cells:***
Adherent breast cancer cell lines were treated/transfected with specified reagents for two days following which $1x10^5$ cells/35mm dish were seeded in methylcellulose. After 48 hrs, cells were harvested from some dishes and subjected to immunoblotting for the specified proteins. Parallel dishes were allowed to form spheres; graph represents spheres formed/20 fields at the end of one week, error bars represent SEM:

C and D) MDAMB231 and BT 474 ($1x10^5$) cells treated with DMSO (vehicle control) or 10 µM AMPK inhibitor Compound C, n=3.

E) MDAMB231 cells were transfected with control siRNA or siRNA targeting AMPKα2 (Cell Signalling), n=4.

F) MDAMB231 cells transfected with a single shRNA construct targeting AMPKα2 (seq#4 from Origene), or scrambled shRNA, were sorted twice for high RFP expressers (shRNA construct encodes for RFP), n=3.

G) Adherent BT 474 cells ($1x10^5$) stably expressing a pool of shRNA targeting AMPK α2, or scrambled vector, n=3.

H and I) ***Effect of overexpression of WT vs S116A mutant of PEA15 on sphere formation by breast cancer cells.***
MDAMB231 cells (H) and BT 474 cells (I) stably transfected with CSCG-WT-flag-PEA15 or CSCG-S116A-flag-PEA15 cells ($1x10^5$) were seeded in methylcellulose. After 7 days, spheres were counted and thereafter spheres/cells were harvested from the same dishes and subjected to immunoblotting for the specified proteins. Graph represents spheres formed/20 fields at the end of one week. Error bars represent SEM; n=3.
