# Supplementary material for: Identification of a novel AMPK-PEA15 axis in the anoikis-resistant growth of mammary cells
Source: Breast Cancer Res. 2014 Aug 6;16:420. doi: 10.1186/s13058-014-0420-z (PMC4303232; doi:10.1186/s13058-014-0420-z)
Supplement: Supplementary file 5 — Additional file 5: Supplementary Figure S5.(PDF 127 KB) [file 13058_2014_420_MOESM5_ESM.pdf]

Supplementary Figure S5

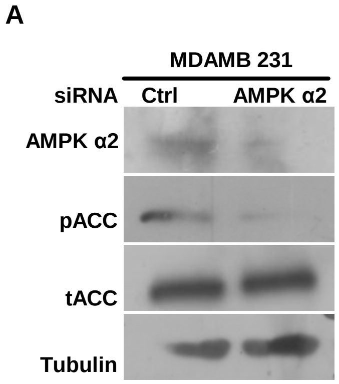

Supplementary Figure S5

A) MDAMB231 cells were transfected with control siRNA or siRNA targeting AMPK α2 and two days post transfection subjected to immunoblotting for the specified proteins.
