# Supplementary figures and images for: Identification of a novel AMPK-PEA15 axis in the anoikis-resistant growth of mammary cells
Source: Breast Cancer Res. 2014 Aug 6;16:420. doi: 10.1186/s13058-014-0420-z (PMC4303232; doi:10.1186/s13058-014-0420-z)

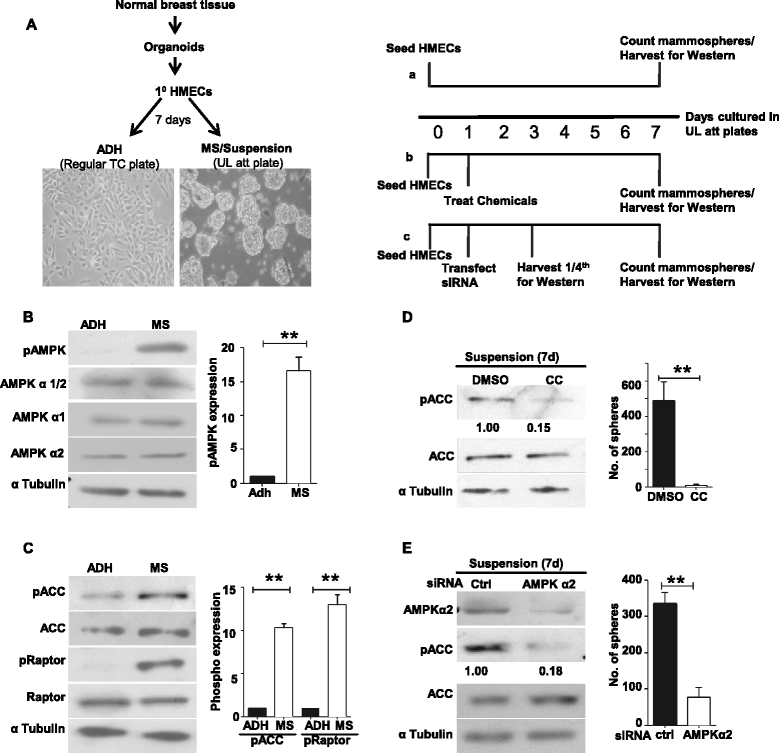

Supplement: Supplementary file 6 — Authors’ original file for figure 1 [file 13058_2014_420_MOESM6_ESM.gif]

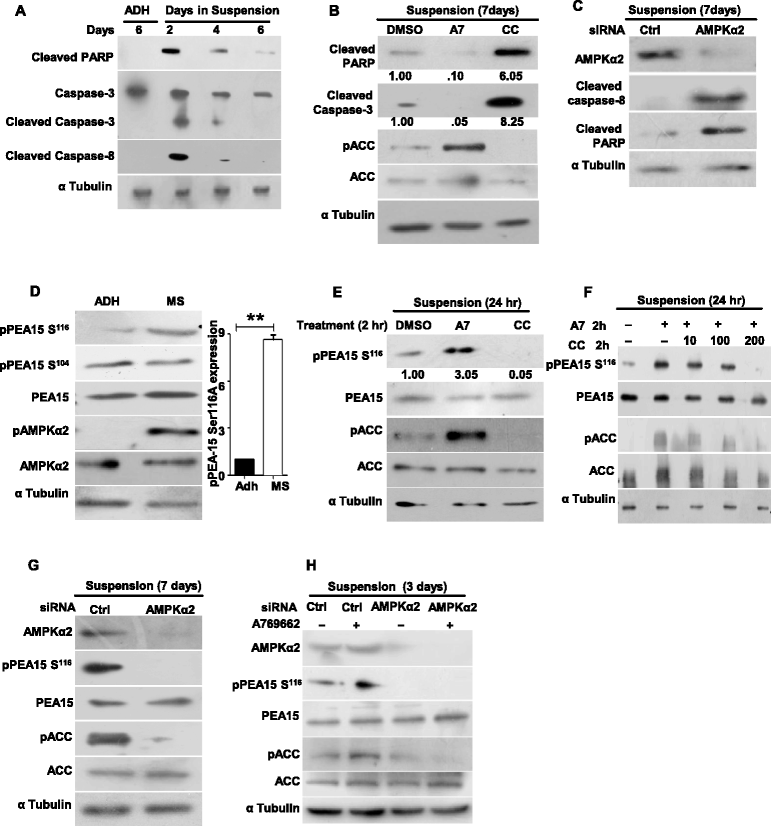

Supplement: Supplementary file 7 — Authors’ original file for figure 2 [file 13058_2014_420_MOESM7_ESM.gif]

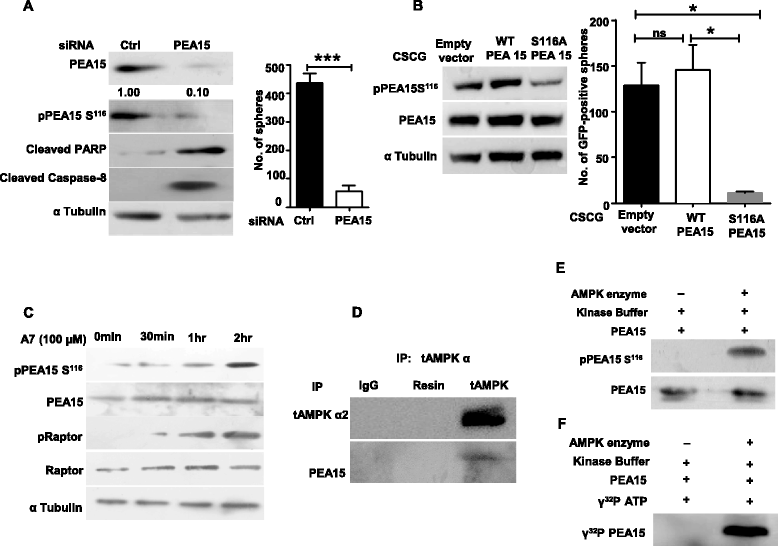

Supplement: Supplementary file 8 — Authors’ original file for figure 3 [file 13058_2014_420_MOESM8_ESM.gif]

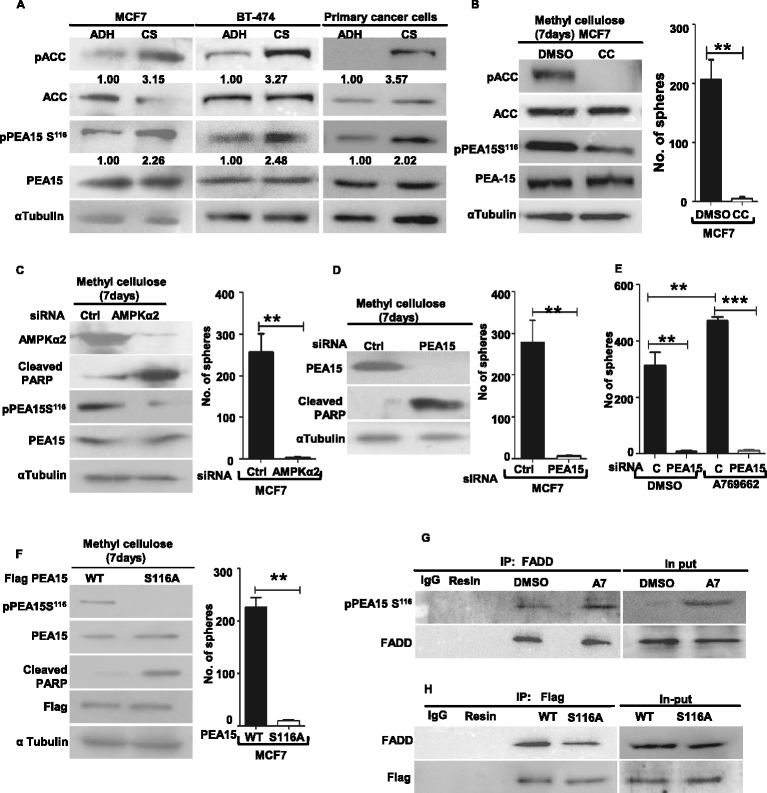

Supplement: Supplementary file 9 — Authors’ original file for figure 4 [file 13058_2014_420_MOESM9_ESM.gif]

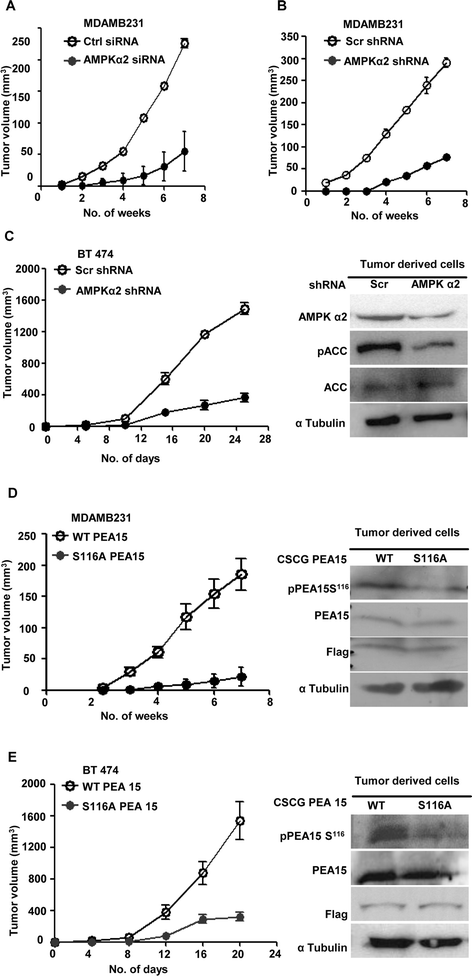

Supplement: Supplementary file 10 — Authors’ original file for figure 5 [file 13058_2014_420_MOESM10_ESM.gif]

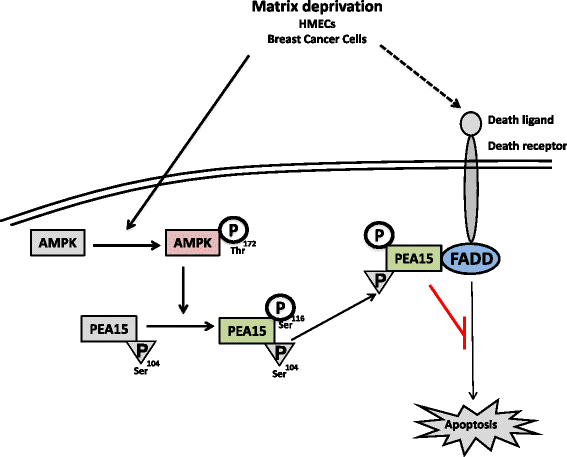

Supplement: Supplementary file 11 — Authors’ original file for figure 6 [file 13058_2014_420_MOESM11_ESM.gif]
